# Comparative assessment of direct absorption solar collector performance in different climates

**DOI:** 10.1038/s41598-023-48780-4

**Published:** 2023-12-04

**Authors:** Mohammad Mahdi Heyhat, Mohammed Qasim Jawad Abbood, Jabraeil Ahbabi Saray, Abolghasem Mokhtari Ardekani

**Affiliations:** 1 Faculty of Mechanical Engineering, Tarbiat Modares University, Tehran, 1411713116 Iran; 2 Iranian Mines and Mining Industries Development and Renovation Organization (IMIDRO), Tehran, Iran

**Keywords:** Climate change, Climate sciences, Environmental sciences, Energy science and technology, Nanoscience and technology

## Abstract

Energy supply and environmental protection by reducing pollutants are among the main challenges these days. As a clean and sustainable source, solar energy is capable of generating thermal and electrical power. In this regard, Iraq is one of the regions with high solar energy harvesting potential. A numerical model was developed and validated by experimental findings in MATLAB software. This model, which also included geometrical and optical characteristics, was developed using information from four cities representative of different climates in Iraq: Baghdad, Samawa, Mosul, and Al-Qa'im. This study examined the effects of climate on the performance of direct absorption parabolic solar collectors used for energy production in Iraq. According to the results, solar collectors in Samaveh provide the highest thermal energy efficiency (up to 66.5%). Even thoth, the highest exergy efficiency is found in Al-Qa'im (36.21%). From an environmental point of view, the collector in Al-Qa'im has the highest CO_2_ mitigation (2.73 kg per m^2^ of collector) every year. As compared to other cities, Al-Qa'im and Samawah have a high thermal efficiency and solar intensity, which can lead to more water and energy savings.

## Introduction

The rising demand for clean energy and protecting the environment by reducing pollutants, especially greenhouse gases, are among the main challenges in today’s world. In this context, the investigation of renewable energy resources is of great importance from an environmental perspective^1^.

Among different renewable energies such as wind, geothermal, and tide, solar energy has attracted the attention of many countries due to its unlimited accessibility and stability^2^. Several systems, including parabolic troughs, Fresnel, and flat collectors, can convert solar radiation into heat. Other systems such as photovoltaic panels have also been developed to convert solar energy directly into to electricity^3,4^.

Angappan et al.^5^ have proposed that combining a solar still and solar box cooker can increase the system's absorption area and water production. According to the results, 3.9 L/m^2^ of water was produced by the passive solar still (PSS), and 5.5 L/m^2^ of water was produced by the active solar still (ASS). Moreover, the cost per liter for PSS and ASS was approximately 0.0101 $ and 0.0091 $, respectively. In addition, compared to the PSS, the ASS was able to reduce CO_2_ emissions by 41%. The effectiveness of solar stills was examined by Panchal et al.^6^ Using evacuated tubes, perforated fins, and pebbles. Considering the results of the study, it was determined that the costs of modified solar still (MSS) were 0.0051 $/L and that of conventional solar still (CSS) were 0.0056 $/L. In addition, MSS was able to reduce CO_2_ emissions by about 2.44 times more than CSS.

A study was presented by Shoeibi et al.^7^ demonstrates the possibility of improving solar still performance with the integration of photovoltaic panels, heat pipes, and thermoelectric generators into a modified single-slope solar still. According to the results of the study, the highest amount of hourly energy produced by conventional solar still and solar still by heat pipes, water cooling and thermoelectric generators (SS-HP-WT) was about 68 W, and 75 W, respectively. Also, conventional photovoltaic, and SS-HP-WT had a cost per unit of 0.061, and 0.147$/kWh, respectively. For the purpose of converting power within the ORC and producing electricity, a study by Gomma et al.^8^ used a hybrid system that utilized waste heat recovery (WHR) coupled with a solar field. WHR was taken from flue gases of cement industry rotary kilns. Parabolic-Trough Solar Collectors (PTSCs), operating with R245fa, were used in the solar field. Based on the study results, the proposed system was capable of generating 360 kW of electricity required to operate a cement plant. The system was also expected to payback time of 3.75 years, with annual savings of $280,000.

Solar collectors raise the fluid temperature. They are categorized into concentrating and non-concentrating classes^9^. The outlet temperature of non-concentrating collectors is generally lower than concentrating ones. Thus, concentrating collectors such as solar dishes or parabolic collectors are more suitable for applications requiring high temperatures.

Naturally, there is a direct relationship between the efficiency of solar collectors and incident irradiation^10^. Additionally, weather conditions such as sunny hours, ambient temperature, and wind velocity affect collector efficiency. Since every geographical zone has its unique weather, it is vital to determine suitable geographical positions. Global climate can be classified into five groups: tropical, temperate, dry, mountainous, and continental^11^. Many researchers have examined the effect of weather conditions on solar collector performance^12,13^. Moreover, solar collectors can be analyzed based on the second law of thermodynamics, i.e., exergy analysis, to indicate the maximum work gained from the system-environment interaction until reaching equilibrium. The parameter of exergy can be also employed for environmental and economic analyses of the system^14^. Some works have considered parameters such as thermal efficiency, outlet temperature, exergy, and energy, as well as economical and environmental considerations, for the optimization of solar collectors. Some of the studies done in this field are reviewed in the following.

An efficient quadruple hybrid system configuration was developed by Zahedi et al.^15^ in order to recover waste heat and enhance efficiency. A total of 327,160 kW of electricity can be generated by this system along with 627,000 tons of carbon dioxide that can be captured and converted into methane fuel. Another study by Zahedi et al.^16^ Discusses the way in which zero-energy buildings can be designed in new cities. A comparison of different methods is conducted to find the most optimal method of home design and energy systems.

Tiwari et al.^17^ analyzed a photovoltaic system combined with a solar water distillation system under various weather conditions in Delhi, India considering environmental and energy-economic issues. Their study revealed the appropriate electricity generation potential of the PCVT-FPC system at sunny hours and supplying daily freshwater. Kaliskan^18^ performed the exergy, energy, and environmental analyses of the use of biomass, solar and electrical power for building heating purposes at eight different reference temperatures. Results showed the solar system as the most optimum and stable option with CO_2_ emissions of 0.1599 kg. Mousavian et al.^11^ investigated the effect of weather on the environmental, economic, exergy and energy performance of a collector in five different cities of Iran. It was concluded that cities with a Mediterranean climate have the highest energy efficiency (71.97%), cities with a continental climate have highest exergy efficiency (22.01%), and cities with a semi-tropical climate were the most efficient in terms of the environmental aspects.

The study by Mujomder et al.^19^ investigated the effects of local climate conditions on the effectiveness of a modified concentrated photovoltaic thermal (CPV/T) collector. Among the other cities, Melbourne is outperforming the rest in terms of the highest energy efficiency for the year (23.75%), while Sydney has the lowest (20.63%). It was found that the average efficiency of collectors in Adelaide and Sydney was 22:45 and 21.70%, respectively, which was the highest and lowest total efficiency, respectively. Due to Melbourne's high solar irradiation in December and Sydney's lowest solar exposure in July, the PV efficiency in December was 0.48 percent higher in Melbourne than in Sydney.

In the Ahliouati et al. study^20^, hybrid photovoltaic and air collectors were used to examine energy performance in El Jadida, Morocco. It is estimated that the daily average efficiency of the PV module is 30.22, and 52.14 for the hybrid air collector (PV/T-Air). Calculations were based on the values for a sunny day in July on the site of El Jadida city.

Herez et al.^21^ explored the performance of a hybrid solar collector in three countries with various climate conditions: Lebanon (temperate), France (temperate), and the United Arab Emirates (hot). The total annual energy generated by PV and TEG were 44.7MWh and 1.8MWh for Lebanon, 26.5MWh and 0.8MWh for France, and 49.2MWh and 1.9MWh for the UAE, respectively. The CO_2_ emission of these countries was 81.4, 5.7, and 79.5 ton, respectively. Ahbabi and Heyhat^22^ conducted thermal modeling and environmental, energy, exergy, and economic analyses for a direct absorption solar collector. Their study aimed to optimize the energy and water consumption of the collector by examining the effective parameters and different nanofluids. They revealed that utilization of CuO + MWCNT/water, MWCNT/water, and CuO/water nanofluids led to saving 40.44GJ, 39.01GJ, and 30.8GJ energy, respectively. The mentioned nanofluids also resulted in water saving of 59.03, 56.95, and 44.96 m^3^, respectively. In a recent study, Zahedi et al.^23^ investigated the potential for solar energy generation along Iran's south-eastern coast. Based on this analysis, the total amount of electricity can be obtained from suitable places in the region was calculated; it was determined that 37.5% of Makran is suitable for the development of solar farms. Makran region can produce approximately 17,200 GWh of electricity annually, which can contribute to the economic, social, and industrial growth of this region.

In accordance with the literature review, renewable energy is readily available in most Asian countries, including solar, hydro, wind, etc.^24^. As one of these countries, Iraq has an excellent potential for the harvesting of solar energy. Recently, only a limited amount of research has been conducted in Iraq on the performance of parabolic solar collectors, which indicates the need for further investigation of the influence of climate on parabolic collectors. In order to determine the most appropriate location for installing the solar collector, it is necessary to compare the performance of the collector using the local ambient and solar radiation data. As a consequence, the present study examines the effect of climate on the performance of direct absorption parabolic solar collectors used for energy production in Iraq. A number of factors, including energy, efficiency, environmental impact, and water-energy nexus, will be taken into consideration when evaluating the collector's performance. These results can be used to determine the most suitable climate for solar energy systems.

## Materials

**Figure 1:**
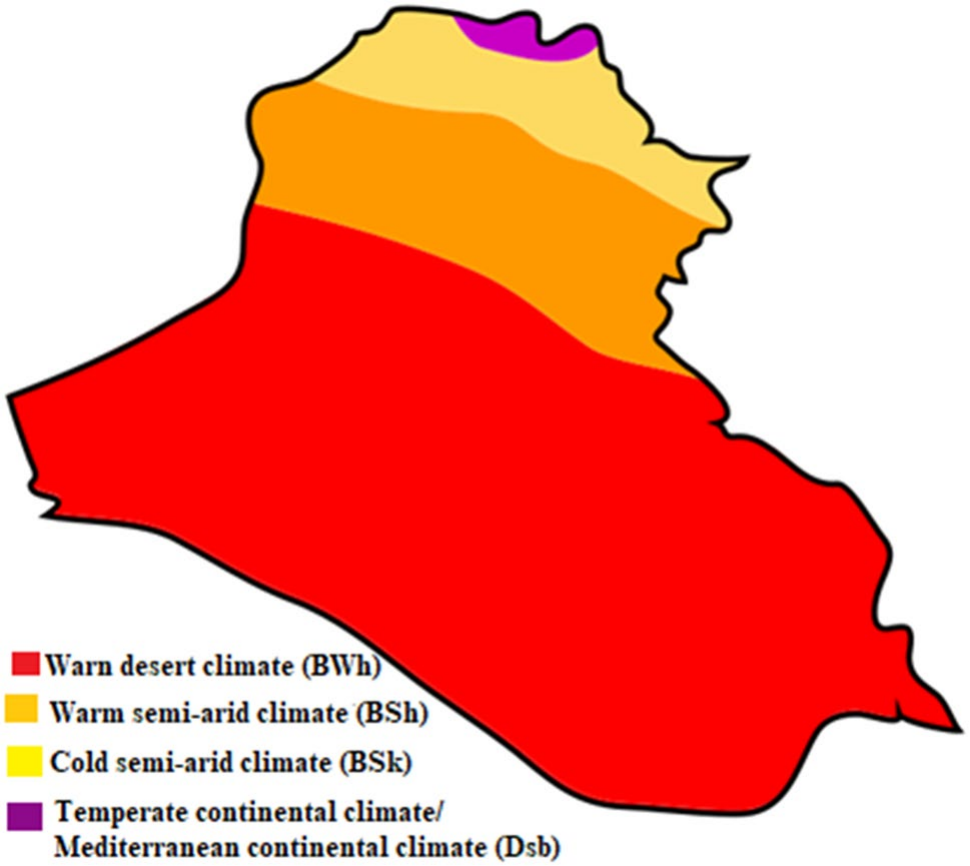
Iraq map of Köppen climate classification^26^.

Iraq is located in southwest Asia at GPS coordinates Latitude of 29$^\circ$ ${37}{\prime}$ and a Longitude of 38$^\circ$ ${48}{\prime}$. The total area of the country sums up to 434,128.00 km^2^^25^. As can be seen from Fig. 1, Iraq has four different climates: temperate continental, warm semi-arid, cold semi-arid, and warm desert. The north of Iraq is mountainous. The middle part of the country is located between two main rivers of Iraq, i.e., Euphrates and Tigris, where the sun mainly shines from the north. The southern part of the country has a sunny climate which is considered one of the regions with the highest solar irradiation. The annual average total solar radiation in southern cities (1978.79 kWh/m^2^/year) is higher than that of northern cities (1755.23 kWh/m^2^/year) (see Table 1).

**Table 1:** The measured meteorological information for four cities^25^.

Location	Latitudes (N)	Elevation (m)	The annual solar radiation kWh/m^2^/year
Baghdad	33°$18^{\prime}$	34.1	1943.73
Samawah	31°$16^{\prime}$	6	1978.79
Mosul	36°$19^{\prime}$	222.9	1755.23
Al-Qaim	33°$02^{\prime}$	615.5	1976.23

**Figure 2:**
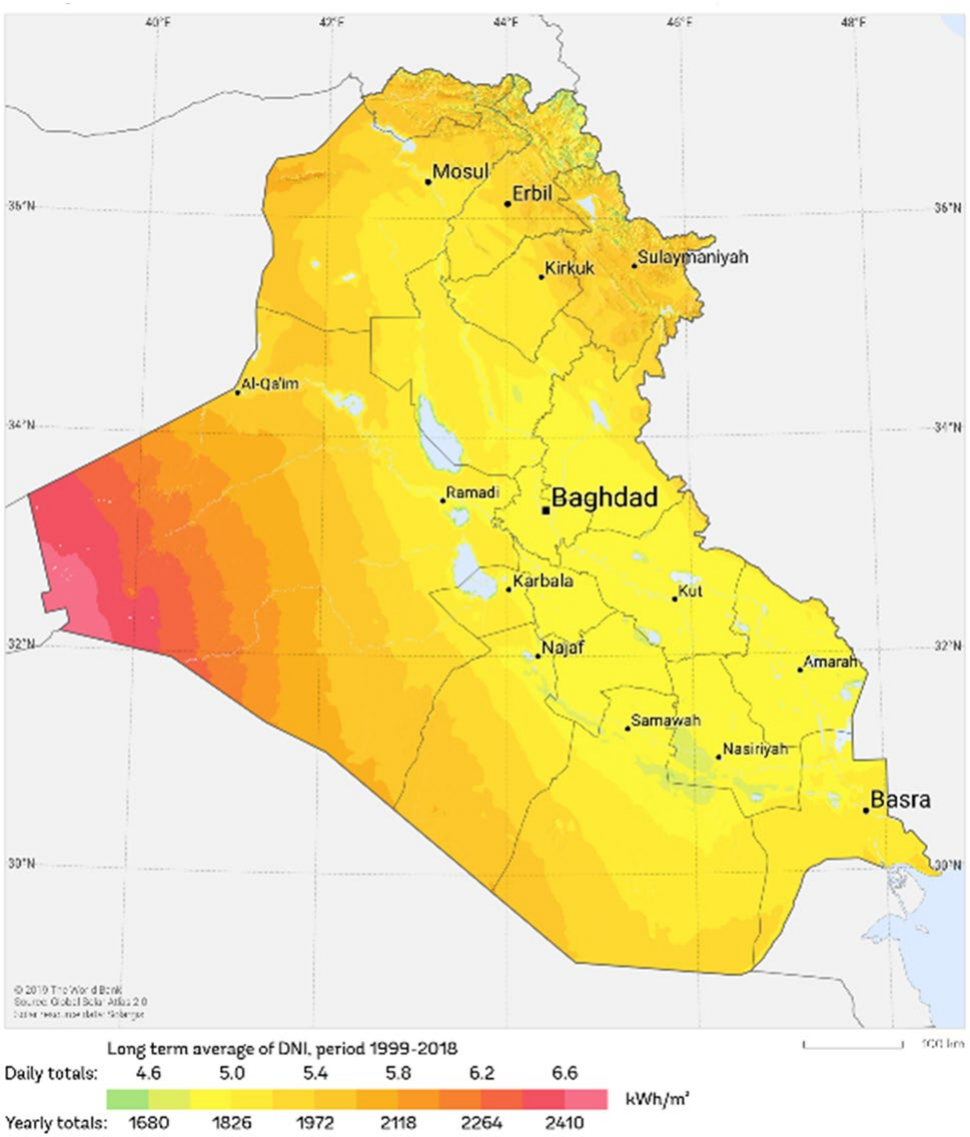
Iraq direct solar irradiation^28^.

Many studies have investigated solar irradiation in Iraq^27^. According to Fig. 2, it has been estimated that the annual solar irradiation in Iraq is in the range of 1680 to 2410kWh/m^2^, and a large part of this irradiation is for the south and west regions of the country. The average solar irradiation in Iraq is higher than the global average^27^.

**Figure 3:**
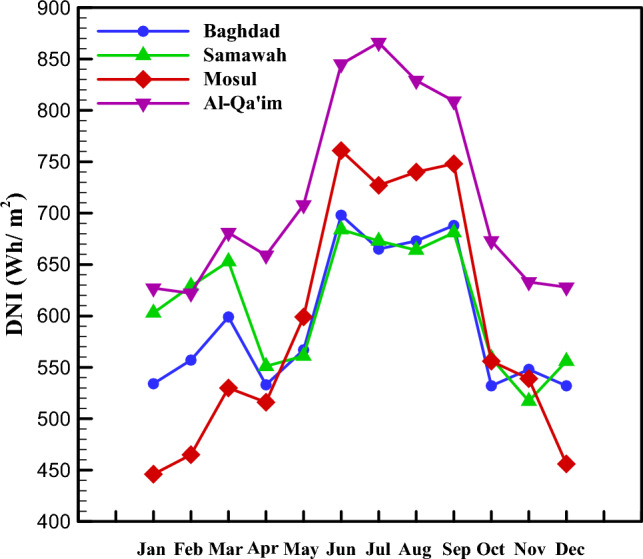
Direct normal irradiation in different climate zones.

**Figure 4:**
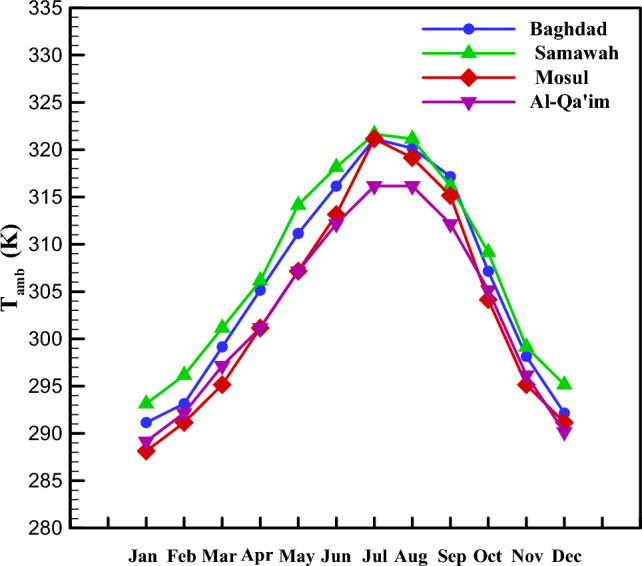
Ambient temperature in different climate zones.

It is better to first examine the solar irradiation intensity and the ambient temperature as climate parameters. The distributions of direct normal solar irradiation and ambient temperature depend on geographical position, season, month, weather, and time of day. Four Iraq cities, i.e., Baghdad, Samawah, Mosul, and Al-Qa'im, with four different climates, were considered. Figure 3 represents the direct normal solar irradiation of these four cities in different months of the year. As seen, Mosul and Al-Qa'im had the maximum and minimum solar irradiations, respectively. The maximum values are related to June, July, August, and September, while the minimum values are for January, February, November, and December. Similarly, the ambient temperature of these four cities is depicted in Fig. 4 in different months of the year. Accordingly, the maximum and minimum ambient temperatures are related to Samawah and Al-Qa'im, respectively. January and July had the maximum and minimum temperatures, respectively. It should be noted that these results are monthly average results measured from 12:00 to 13:00.

**Figure 5:**
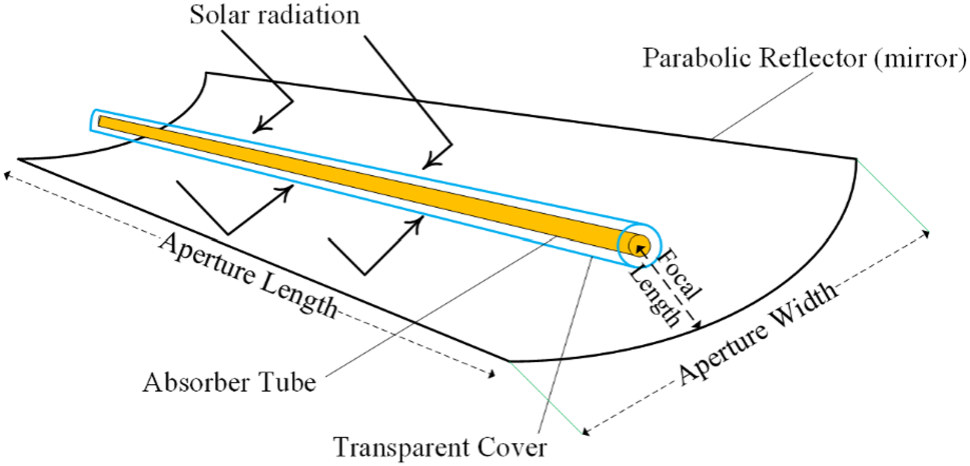
Schematic of DAPTC.

The present study used the collection used by^29^ to investigate the influence of weather variations on energy and energy efficiency as well as environmental effects. A schematic of DAPTC is depicted in Fig. 5. Table 2 also lists the features of the applied collector including its geometrical dimensions, operational conditions, and optical parameters, which were considered in the numerical modeling.

**Table 2:** General characteristics of the DAPTC^30^.

Parameter	Value	Unit	Symbol
Optical properties			
Glass tube emittance	–	0.86	${\upvarepsilon }_{\mathrm{t}}$
Glass tube absorbance	–	0.02	${\mathrm{\alpha }}_{\mathrm{t}}$
Glass tube transmittance	–	0.95	${\uptau }_{\mathrm{t}}$
Tracking and other errors	–	0.9	$\upgamma$
Mirror reflectance	–	0.93	r_m_
Glass cover emittance	–	0.86	${\upvarepsilon }_{\mathrm{c}}$
Glass cover absorbance	–	0.02	${\mathrm{\alpha }}_{\mathrm{c}}$
Glass cover transmittance	–	0.95	${\uptau }_{\mathrm{c}}$
Geometrical properties			
Width	m	0.7	G
Length	m	1.5	L
Focal length	m	0.175	F
Glass tube outer diameter	m	0.026	D_t-o_
Glass tube inner diameter	m	0.022	D_t-in_
Glass cover outer diameter	m	0.06	D_c-o_
Glass cover inner diameter	m	0.05	D_c-in_
Operating conditions			
Nanofluid inlet temperature	$^\circ{\rm C}$	30	T_in_
Air temperature	$^\circ{\rm C}$	26	T_a_
Nanofluid flow rate	LPH	60	$\dot{\mathrm{V}}$
Solar irradiance	$\frac{\mathrm{W}}{{\mathrm{m}}^{2}}$	900	I

## Methodology

The thermal modeling of the direct-absorption parabolic solar collector employed in the present study is thoroughly described in^22^. For modeling, the optical, geometrical, and operating properties of the collector as well as the weather conditions are as input data. The outputs are energy and exergy efficiencies, fluid outlet temperature, CO_2_ mitigation, and embodied energy and water.

### Thermal modeling

Equation (1) shows the optical efficiency of the collector^31^.

1 $${\upeta }_{{{\text{opt}}}} = {\text{r}}_{{\text{m}}} {{ \upgamma \upalpha }}_{{\text{c}}} {\text{ K}}\left( {\uptheta } \right){\text{ X}}_{{{\text{end}}}}$$

The incident angle modifier K (θ) can be estimated using the following formula^32^.

2 $${\text{K}}\left( {\uptheta } \right) = { }\frac{1}{\cos \theta } \times \left( {\cos \theta + 0.000884\theta - 0.00005369\theta^{2} } \right)$$

${\mathrm{X}}_{\mathrm{end}}$ is the end loss factor that depends on collector length, focal length, and incident angle^10^:

3 $${\mathrm{X}}_{\mathrm{end}}=1-\frac{\mathrm{F}}{\mathrm{L}}\mathrm{tan\theta }$$

**Figure 6:**
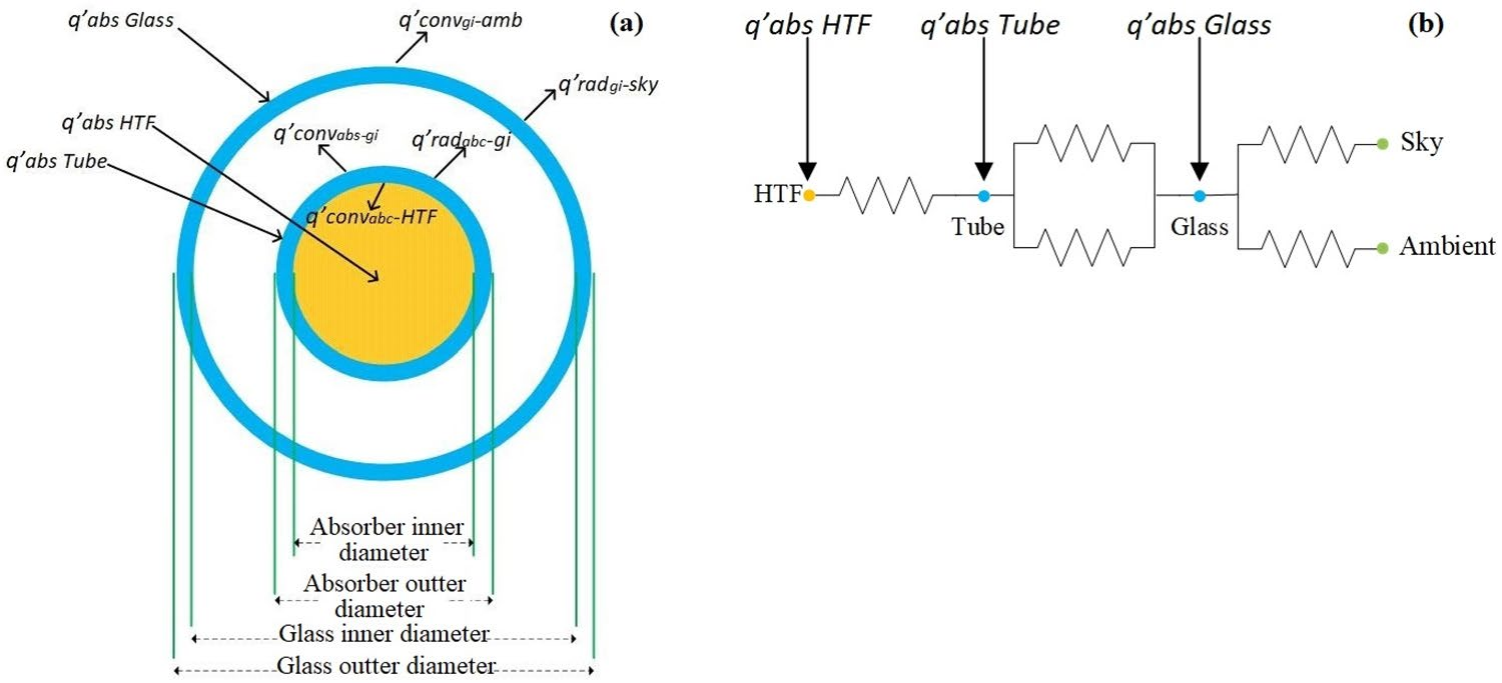
(**a**) Cross-section of glass tube diameter and (**b**) thermal resistance.

Different types of heat transfer in the parabolic collector are needed to extract the governing thermal equations. Figure 6 presents the cross-section of glass tube diameter and thermal resistance of the system^22^.

4 $${\dot{\mathrm{Q}}}_{\mathrm{c}-\mathrm{abs}}+{\dot{\mathrm{Q}}}_{\mathrm{t}-\mathrm{c}}^{\mathrm{conv}}+{\dot{\mathrm{Q}}}_{\mathrm{t}-\mathrm{c}}^{\mathrm{rad}}={\dot{\mathrm{Q}}}_{\mathrm{c}-\mathrm{sa}}^{\mathrm{conv}}+{\dot{\mathrm{Q}}}_{\mathrm{c}-\mathrm{s}}^{\mathrm{rad}}$$

5 $${\dot{\mathrm{Q}}}_{\mathrm{t}-\mathrm{abs}}={\dot{\mathrm{Q}}}_{\mathrm{t}-\mathrm{c}}^{\mathrm{conv}}+{\dot{\mathrm{Q}}}_{\mathrm{t}-\mathrm{c}}^{\mathrm{rad}}+{\dot{\mathrm{Q}}}_{\mathrm{t}-\mathrm{f}}^{\mathrm{conv}}$$

6 $${\dot{\mathrm{Q}}}_{\mathrm{f}-\mathrm{abs}}+{\dot{\mathrm{Q}}}_{\mathrm{t}-\mathrm{f}}^{\mathrm{conv}}=\dot{\mathrm{m}} {\mathrm{c}}_{\mathrm{p}} ({\mathrm{T}}_{\mathrm{i}+1}-{\mathrm{T}}_{\mathrm{i}})$$

The rate of heat absorbed by the heat transfer fluid and glasses are shown in blew^33^.

7 $${\dot{\mathrm{Q}}}_{\mathrm{c}-\mathrm{abs}}={\upeta }_{\mathrm{opt}}\mathrm{ I}$$

8 $${\dot{\mathrm{Q}}}_{\mathrm{t}-\mathrm{abs}}={\dot{\mathrm{Q}}}_{\mathrm{c}-\mathrm{abs}} \left(\frac{{\mathrm{\alpha }}_{\mathrm{t}}{\uptau }_{\mathrm{c}}}{{\mathrm{\alpha }}_{\mathrm{c}}}\right)$$

9 $${\dot{\mathrm{Q}}}_{\mathrm{f}-\mathrm{abs}}={\dot{\mathrm{Q}}}_{\mathrm{t}-\mathrm{abs}} \left(\frac{{\mathrm{\alpha }}_{\mathrm{f}}{\uptau }_{\mathrm{t}}}{{\mathrm{\alpha }}_{\mathrm{t}}}\right)$$

where *I* (W/m^2^) is the radiation intensity. The absorption coefficient of nanofluids ${\mathrm{\alpha }}_{\mathrm{f}}$ relies on the type of nanofluids and their volume fractions. For Al_2_O_3_/water and MWCNT/water nanofluids, it can be used from^29^.

The radiation heat transfer rate is calculated as follows^34^:

10 $${\dot{\mathrm{Q}}}_{\mathrm{t}-\mathrm{c}}^{\mathrm{rad}}=\frac{\uppi {\mathrm{ D}}_{\mathrm{out}-\mathrm{c}}\upsigma \left({\mathrm{T}}_{\mathrm{t}}^{4}-{\mathrm{T}}_{\mathrm{c}}^{4}\right)}{\frac{1}{{\upvarepsilon }_{\mathrm{t}}}+\frac{1-{\upvarepsilon }_{\mathrm{c}}}{{\upvarepsilon }_{\mathrm{c}}} \frac{{\mathrm{D}}_{\mathrm{t}-\mathrm{o}}}{{\mathrm{D}}_{\mathrm{c}-\mathrm{in}}}}$$

where ${\upvarepsilon }_{\mathrm{c}}$ and ${\upvarepsilon }_{\mathrm{t}}$ are emission coefficients of cover and tube and ${\mathrm{T}}_{\mathrm{c}} (\mathrm{K})$ and ${\mathrm{T}}_{\mathrm{t}} \left(\mathrm{K}\right)$ are their temperatures, respectively. Assuming to vacuum state between the annulus, the heat transfer coefficient is specified by Eq. (11)^34^.

11 $${\mathrm{h}}_{\mathrm{t}-\mathrm{c}}=\frac{\uplambda }{\frac{{\mathrm{D}}_{\mathrm{t}-\mathrm{o}}}{2\mathrm{ln}\left(\frac{{\mathrm{D}}_{\mathrm{c}-\mathrm{in}}}{{\mathrm{D}}_{\mathrm{t}-\mathrm{o}}}\right)}+\mathrm{bq }(\frac{{\mathrm{D}}_{\mathrm{t}-\mathrm{o}}}{{\mathrm{D}}_{\mathrm{c}-\mathrm{in}}}+1)}$$

$\uplambda (\frac{\mathrm{w}}{\mathrm{m}.\mathrm{ K}})$ is thermal conductivity. For air at 300 $^\circ{\rm C}$ and pressure 0.03 Pa, q, and b the mean-free-path collisions of a molecule and interaction coefficient are equal to 88.67 cm and 1.571^34^, respectively. The rate of heat loss from tube to surrounding air can be calculated as follows^35^.

12 $${\dot{\mathrm{Q}}}_{\mathrm{c}-\mathrm{sa}}^{\mathrm{conv}}=\uppi {\mathrm{D}}_{\mathrm{c}-\mathrm{o}} {\mathrm{h}}_{\mathrm{c}-\mathrm{sa}} \left({\mathrm{T}}_{\mathrm{c}}-{\mathrm{T}}_{\mathrm{sa}}\right)$$

In the above relation ${\mathrm{h}}_{\mathrm{c}-\mathrm{sa}}$ is the heat transfer coefficient which determined by the Nusselt number^35^:

13 $$\begin{gathered} {\text{Nu}}_{{{\text{air}}}} = \frac{{{\text{h}}_{{{\text{c}} - {\text{sa}}}} {\text{ D}}_{{{\text{c}} - {\text{o}}}} }}{{{\uplambda }_{{{\text{air}}}} }} = \left\{ \begin{gathered} 0.4 + 0.54 {\text{Re}}_{{{\text{air}}}}^{0.52} \;\;0.1 < {\text{Re}}_{{{\text{air}}}} < 1000 \hfill \\ 0.3 {\text{Re}}_{{{\text{air}}}}^{0.6} \quad \quad \quad \;\;1000< {{\text{Re}}_{{{\text{air}}}} } < 50000 \hfill \\ \end{gathered} \right. \hfill \\ {\text{Nu}}_{{\text{D}}} = 4.36\quad \quad {\text{Re}} < 2300 \hfill \\ \end{gathered}$$

In the following equation, the rate of heat loss from the collector tube to the sky is calculated^36^.

14 $${\dot{\mathrm{Q}}}_{\mathrm{c}-\mathrm{s}}^{\mathrm{rad}}=\uppi {\mathrm{D}}_{\mathrm{c}-\mathrm{o}}\upsigma {\upvarepsilon }_{\mathrm{c}} \left({\mathrm{T}}_{\mathrm{c}}^{4}-{\mathrm{T}}_{\mathrm{sky}}^{4}\right)$$

where ${\mathrm{T}}_{\mathrm{sky}} (K)$ is sky temperature^36^:

15 $${\mathrm{T}}_{\mathrm{sky}}=0.0553 {\mathrm{T}}_{\mathrm{sa}}^{1.5}$$

In this study, water is used as base-fluid and aluminum oxide (Al_2_O_3_) and multi-walled carbon nanotube (MWCNT) as nanoparticles. The physical properties of nanomaterials are shown in Table 3.

**Table 3:** Properties of the used nanomaterials.

Nanoparticle	$\uprho$ (kg/m^3^)	${\mathrm{C}}_{\mathrm{P}}$ (J/kg)	K (W/m K)
Al_2_O_3_^37^	3970	773	40
MWCNT^22^	2100	3000	791

### Energy and exergy analysis

The rate of useful energy depends on outlet and inlet fluid temperature difference as well as mass nanofluid flow rate and heat capacity of nanofluid^38^. The thermal efficiency of the solar collector is described as Eq. (17).

16 $${\dot{\mathrm{Q}}}_{\mathrm{u}}=\dot{\mathrm{m}} {{\mathrm{C}}_{\mathrm{p}}}_{\mathrm{nf}} \left({\mathrm{T}}_{\mathrm{out}}-{\mathrm{T}}_{\mathrm{in}}\right)$$

17 $${\upeta }_{\mathrm{th}}=\frac{{\dot{\mathrm{Q}}}_{\mathrm{u}}}{\mathrm{IGL}}$$

Considering the solar collector as a control volume for exergy analysis, the exergy balance can be written as following^39^:

18 $${{\dot{\mathrm{Ex}}}_{\mathrm{s}}+{\dot{\mathrm{Ex}}}_{\mathrm{in}}-{\dot{\mathrm{Ex}}}_{\mathrm{out}}-\dot{\mathrm{Ex}}}_{\mathrm{loss}}-{\dot{\mathrm{Ex}}}_{\mathrm{d}}=0$$

In the above, ${\dot{\mathrm{Ex}}}_{\mathrm{s}}$ is the exergy of the sun, ${\dot{\mathrm{Ex}}}_{\mathrm{d}}$ is exergy destruction, and ${\dot{\mathrm{Ex}}}_{\mathrm{loss}}$ is the exergy loss in the collector. By employing the Petela Equation, one can calculate the magnitude of the exergy from solar irradiance^39^.

19 $${\dot{\mathrm{Ex}}}_{\mathrm{s}}=\mathrm{IGL}\cdot \left[1-\frac{4}{3}\cdot \left(\frac{{\mathrm{T}}_{\mathrm{sa}}}{{\mathrm{T}}_{\mathrm{s}}}\right)+\frac{1}{3}\cdot {\left(\frac{{\mathrm{T}}_{\mathrm{sa}}}{{\mathrm{T}}_{\mathrm{s}}}\right)}^{4}\right]$$

The useful exergy is described as the difference between outlet and inlet flow’s exergy, and it can be calculated by Eq. (20)^22^.

20 $${\dot{\mathrm{Ex}}}_{\mathrm{u}}=\dot{\mathrm{m}} {\mathrm{C}}_{\mathrm{p}} \left({\mathrm{T}}_{\mathrm{out}}-{\mathrm{T}}_{\mathrm{in}}\right)-\dot{\mathrm{m}} {\mathrm{C}}_{\mathrm{p}}{\mathrm{ T}}_{\mathrm{sa}}\mathrm{ ln}\left(\frac{{\mathrm{T}}_{\mathrm{out}}}{{\mathrm{T}}_{\mathrm{in}}}\right)$$

Eventually, Eq. (21) can be used to calculate the exergy efficiency^22^.

21 $${\upeta }_{\mathrm{Ex}}=\frac{{\dot{\mathrm{Ex}}}_{\mathrm{u}}}{{\dot{\mathrm{Ex}}}_{\mathrm{s}}}=1-\frac{{\dot{\mathrm{Ex}}}_{\mathrm{loss}}+{\dot{\mathrm{Ex}}}_{\mathrm{d}}}{{\dot{\mathrm{Ex}}}_{\mathrm{s}}}$$

### Environmental analysis

The CO_2_ mitigation from an economic perspective is an effort to make environmental effects more tangible. The energo-enviro-economic (ENENEC) method involve incentives to reduce the environmental impacts of energy systems^14^.

22 $${\mathrm{x}}_{{\mathrm{co}}_{2}}={\mathrm{y}}_{{\mathrm{co}}_{2}} {\dot{\mathrm{Q}}}_{\mathrm{u}} {\mathrm{t}}_{\mathrm{annual}}$$

In the above relation, ${\mathrm{x}}_{{\mathrm{co}}_{2}}$ indicates the amount of CO_2_ emission (kg), ${\dot{\mathrm{Q}}}_{\mathrm{u}}$ is the useful energy received, and ${\mathrm{t}}_{\mathrm{annual}}$ is the collector’s working time. Based on life-cycle assessment, the amount of yco_2_ varies depending on the type of electricity generation source. This information can be found in Table 4. To calculate CO_2_ mitigation, the difference in CO_2_ emissions from natural gas (a common energy source for power generation in the country) and solar energy can be used to achieve this. The cost of CO_2_ emission by the solar collector can be calculated as follow^14^.

**Table 4:** Some of electricity generation sources with CO_2_ emission values^41^.

System	CO_2_ emission value
Nuclear	0.0242–0:066
Biomass	0.015–0.178
Natural gas	0.011
Solar PV	0.0534–0.032
Coal	0.960–1.050
Biogas	0.011
Fuel cell	0.038
Oil	0.7421–0.778
Solar collector^42^	0.00647

23 $${\mathrm{C}}_{{\mathrm{co}}_{2}}={\mathrm{x}}_{{\mathrm{co}}_{2}} {\mathrm{c}}_{{\mathrm{co}}_{2}}$$

There are differences in the price of carbon emissions $${\mathrm{c}}_{{\mathrm{co}}_{2}}$$ from 13 to 16 ($/ton) in different countries. Many studies have considered the mean value of 14.5 ($/kg)^14,40^.

## Validation

Before presenting the results of the thermal modeling, the model validation should be provided. To this end, the numerical modeling results were compared with the experimental results of Mashhadian et al.^29^. It is evident that the modeling conditions should be fully compatible with experimental conditions (provided in Table 2) so that the comparison can be made. Table 5 shows validation results for the working fluid's thermal efficiency and outlet temperature.

**Table 5:** Verification of the empirical results.

Cases	Nanofluid	G_I_	T_0_	T_in_	V	$\Delta T (K)$			${\upeta }_{\mathrm{th}} (\%)$		
		W/m^2^	K	K	LPH	Exp.^29^	Model	Error (%)	Exp.^29^	Model	Error (%)
1	MWCNT 0.01% /water	834	288.15	293.15	40	280.73	281.29	0.2	44.03	44.92	1.96
2	MWCNT 0.02% /water	828	298.15	393.15	60	279.19	280.58	0.5	52.31	53.58	3.05
3	MWCNT 0.04% /water	867	303.15	313.15	80	278.26	279.92	0.6	55	56.13	2.07
Mean								0.43			2.36
4	Al_2_O_3_ 0.01% /water	856	288.15	293.15	40	277.81	278.64	0.3	26	26.48	1.8
5	Al_2_O_3_ 0.02% /water	814	298.15	393.15	60	276.65	278.31	0.6	30.5	31.26	2.5
6	Al_2_O_3_ 0.04% /water	816	303.15	313.15	80	276.29	278.19	0. 7	37	37.85	2.3
Mean								0. 53			2.2
7	Hybrid 0.01% /water	845	288.15	293.15	40	281.43	282.55	0.4	45	46.44	3.2
8	Hybrid 0.02% /water	846	298.15	393.15	60	279.83	281.50	0.6	55.5	56.8	2.7
9	Hybrid 0.04% /water	856	303.15	313.15	80	278.45	279.84	0.5	61	62.58	2.6
Mean								0.5			2.83

## Results and discussion

According to a previous study^29^, it is concluded that the hybrid nanofluid (Al_2_O_3_-MWCNT) at a glass tube diameter of 2 cm and inlet temperature of 30 $^\circ{\rm C}$ has a higher performance from the energy point of view; therefore, in the following study, these parameters are used. In this section, the energy, exergy, economic, and environmental aspects were analyzed to investigate the effect of climate conditions on the direct absorption parabolic collector performance.

### Energy analysis of direct-absorption parabolic collector

**Figure 7:**
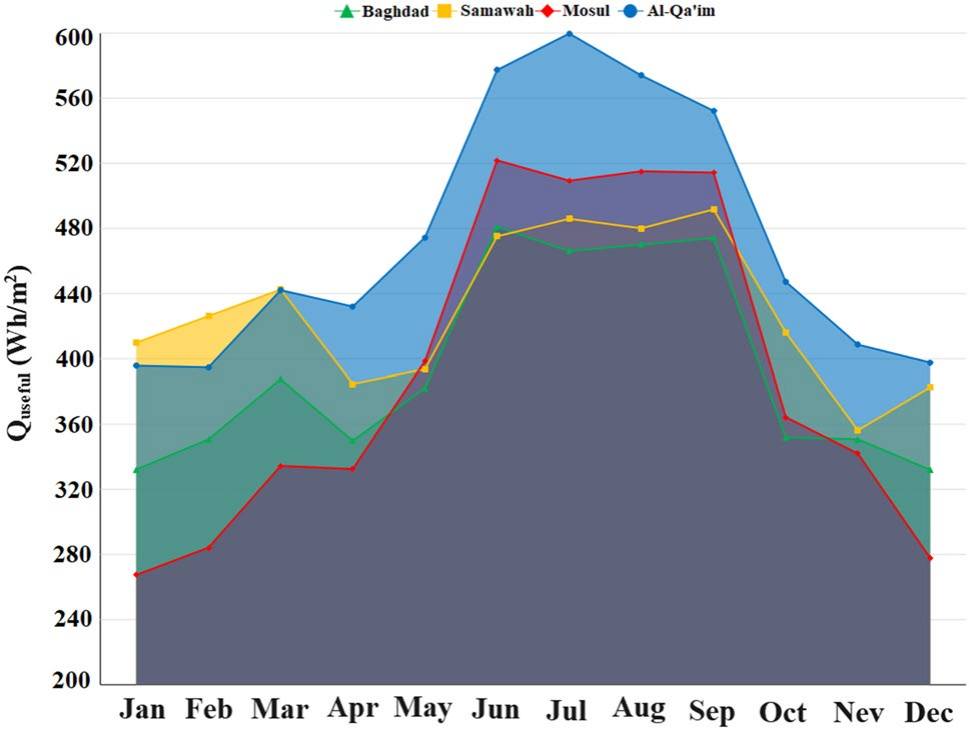
Monthly change in useful energy of the collector.

**Figure 8:**
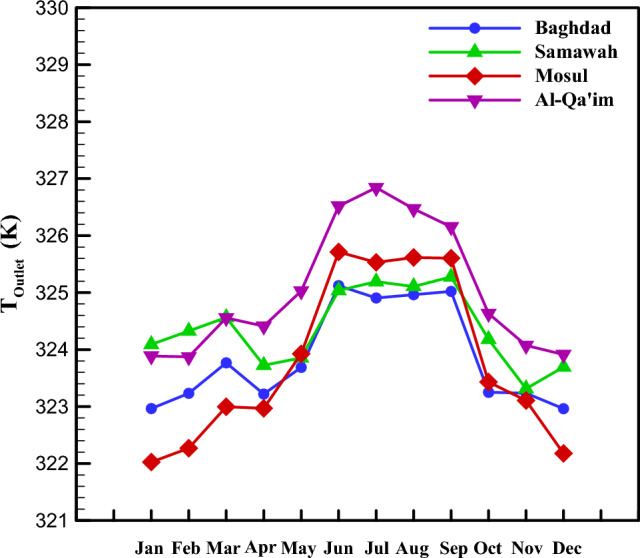
Outlet temperature of DAPTC in the different climatic zones.

As mentioned in Sections “Methodology” and “Materials”, the energy distribution in the system is such that solar energy enters the system as input, a part of which is lost due to thermal and optical losses, and the working fluid absorbs the remaining portion as useful energy. Figure 7 depicts the useful energy received by these four cities in different months. As can be seen, Al-Qa'im city received more useful energy than the others. Overall, June, July, August, and September are the months with higher useful energy. The maximum received energy was for Al-Qa'im city in Jul (599.75 Wh/m^2^), while the minimum value was recorded for Mosul city in January (267.43 Wh/m^2^). Using the amount of useful energy received, one can calculate the collector outlet temperature. Based on Fig. 8, the maximum outlet temperature is obtained in January, July, August, and September for Al-Qa'im and Mosul cities. Al-Qa'im recorded the highest outlet temperature (326.84 K) in July, while Mosul recorded the lowest outlet temperature (322.02 K) in January.

**Figure 9:**
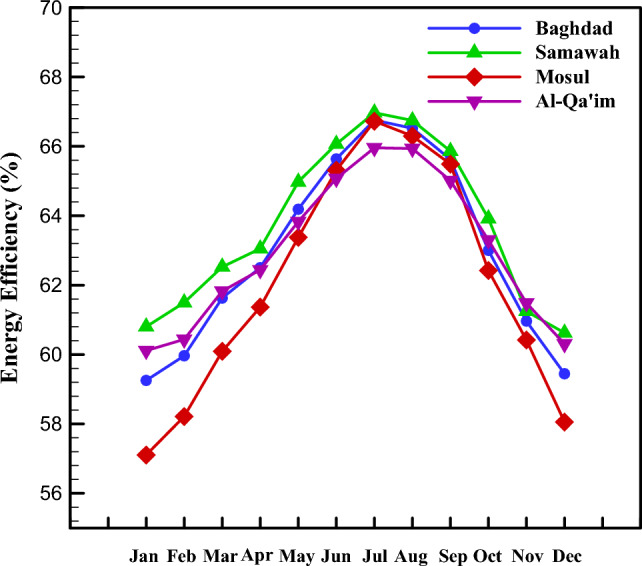
Monthly change in energy efficiency of different cities.

Figure 9 presents the thermal efficiency of the solar collector installed in Baghdad, Samawah, Mosul, and Al-Qa'im cities for different months. As seen, all cities achieved their maximum thermal efficiency in July. Besides, the minimum thermal efficiency was in January. The Samawah, among others, has higher thermal efficiency.

### Exergy analysis of direct-absorption parabolic collector

**Figure 10:**
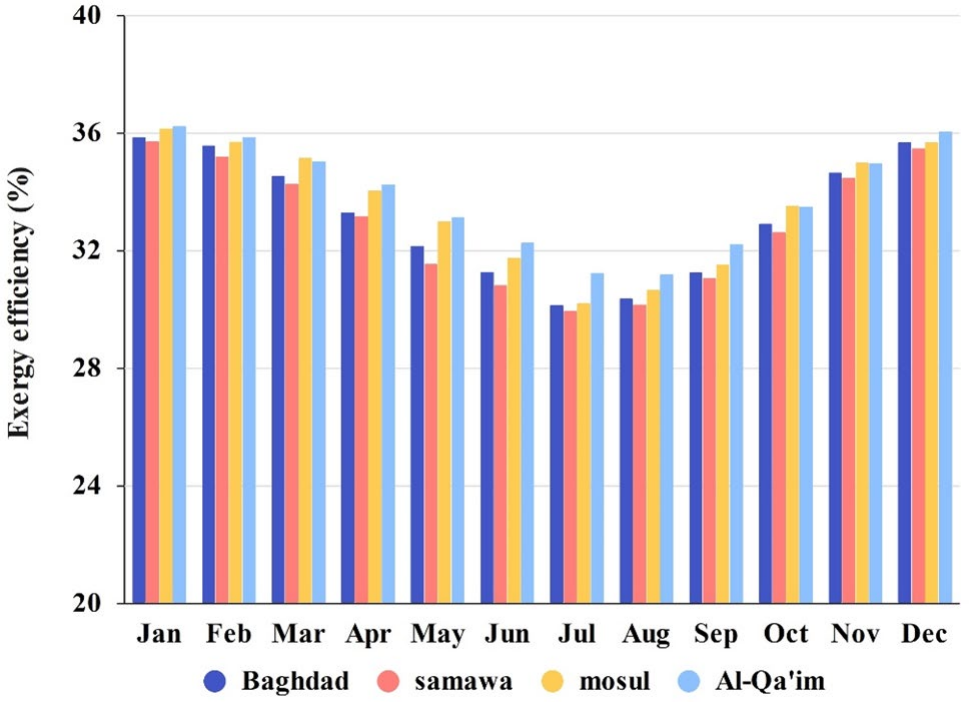
Monthly change in exergy efficiency of different cities.

The same energy analysis can be used in terms of exergy. The exergy balance in the system is such that the solar exergy enters the system, a part of which is lost due to the thermal and optical losses; besides, a part of the exergy is destructed due to friction and irreversibility; and the nanofluid absorbs the remaining as useful energy. Figure 10 shows the exergy efficiency for four cities in different months. As opposed to energy efficiency, the collectors in Mosul and Al-Qa'im had a higher energy efficiency.

Interestingly, the maximum and minimum exergy efficiency of all cities were obtained in January and July, respectively. The reason is the significant influence of ambient temperature on the exergy efficiency, unlike the case for energy. Regarding the maximum temperature in June, July, and August, the temperature difference between the fluid and environment is small. The maximum exergy efficiency (36.21%) was recorded for Al-Qa'im in January, while the minimum exergy efficiency (29.93%) was recorded for Samawah in July.

**Figure 11:**
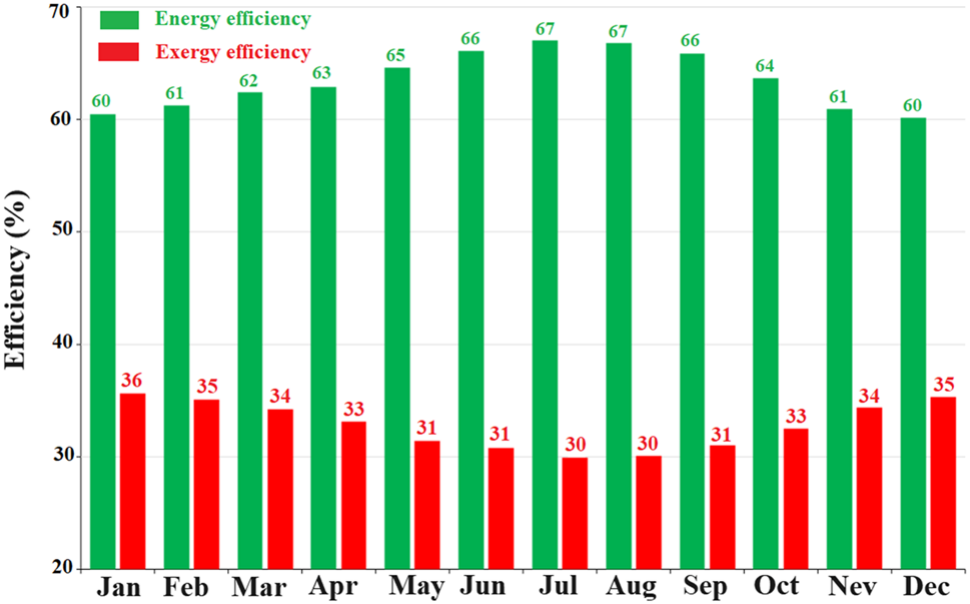
Monthly change in energy-exergy efficiency of Samawah.

Figure 11 represents the energy and exergy efficiency of the solar collector installed in Samawah city during various months. This plot reveals the reverse trend of energy efficiency compared to exergy efficiency. Based on Fig. 11, the maximum energy efficiency was achieved in July and August, while the exergy efficiency was minimal in these months. On the other hand, the energy efficiency in December and January was minimum while the exergy efficiency showed its maximum value.

**Figure 12:**
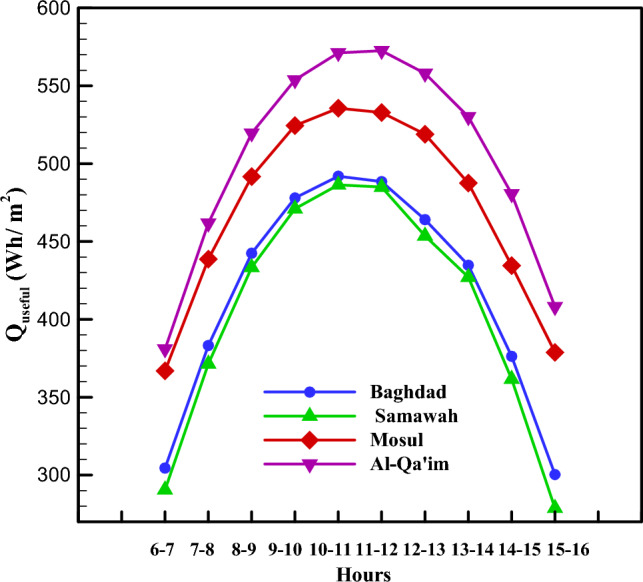
Hourly change in DAPTC energy useful for July.

**Figure 13:**
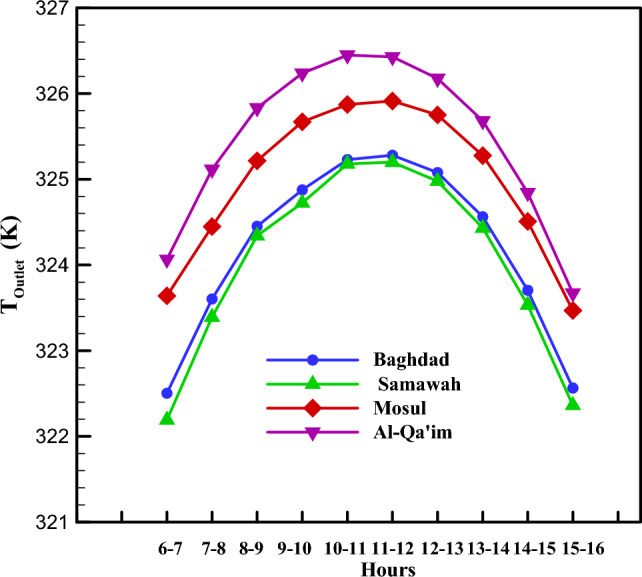
Hourly change in DAPTC outlet temperature for July.

**Figure 14:**
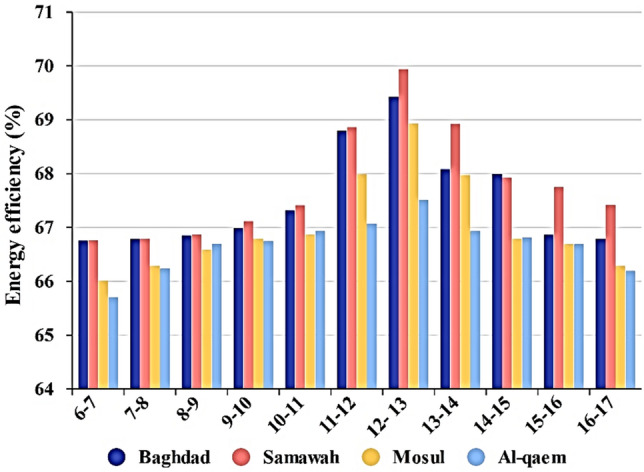
Hourly change in DAPTC energy efficiency for July.

**Figure 15:**
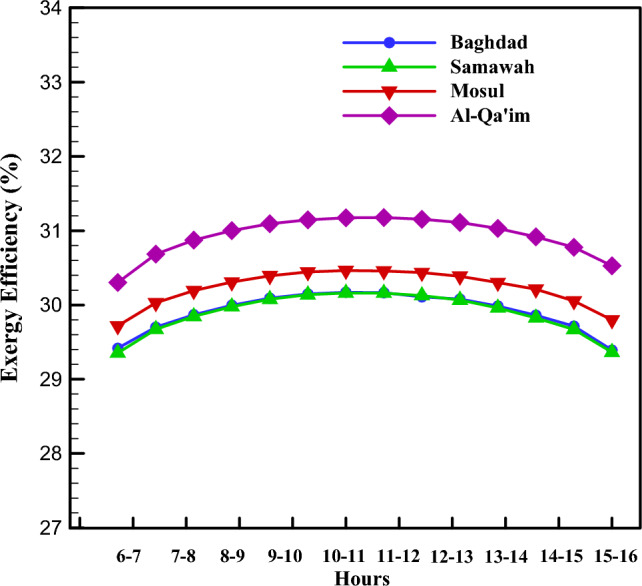
Hourly change in DAPTC exergy efficiency for July.

Figures 7, 8, 9, 10 and 11 illustrate monthly analyses conducted between 12:00 and 13:00. For further investigations, all cities were assessed at all hours of the day in July (see Figs. 12, 13, 14 and 15). Based on Fig. 12, the utilization of collectors in all cities had the maximum useful energy from 9:00 to 13:00. As a result, the outlet temperature of the fluid in this period was higher (see Fig. 13). The maximum values of useful energy and outlet temperature in Al-Qa'im were 572 Wh/m^2^ and 326 K, respectively.

From energy point of view (see Fig. 14), collector efficiency constantly increases from the morning to noon. A dramatic increase in collector efficiency was observed at noon, i.e., 11 a.m.–1 p.m. The reason for this increase corresponds to the higher solar irradiation intensity.

The maximum energy efficiency, 69.9%, was for Samawah city from 12 a.m. to 1 p.m. It can be concluded that Al-Qaim has more solar irradiation intensity and useful energy, but the energy efficiency of Samavah is somewhat higher.

Figure 15 shows the variations in exergy efficiency during the day. Higher exergy efficiencies were recorded from 11:00 to 13:00 in all cities. The Al-Qa'im and Mosul had higher efficiency, respectively. Baghdad and Samawah results were almost the same.

### Environmental and economic-environmental analyses of DAPTC

**Figure 16:**
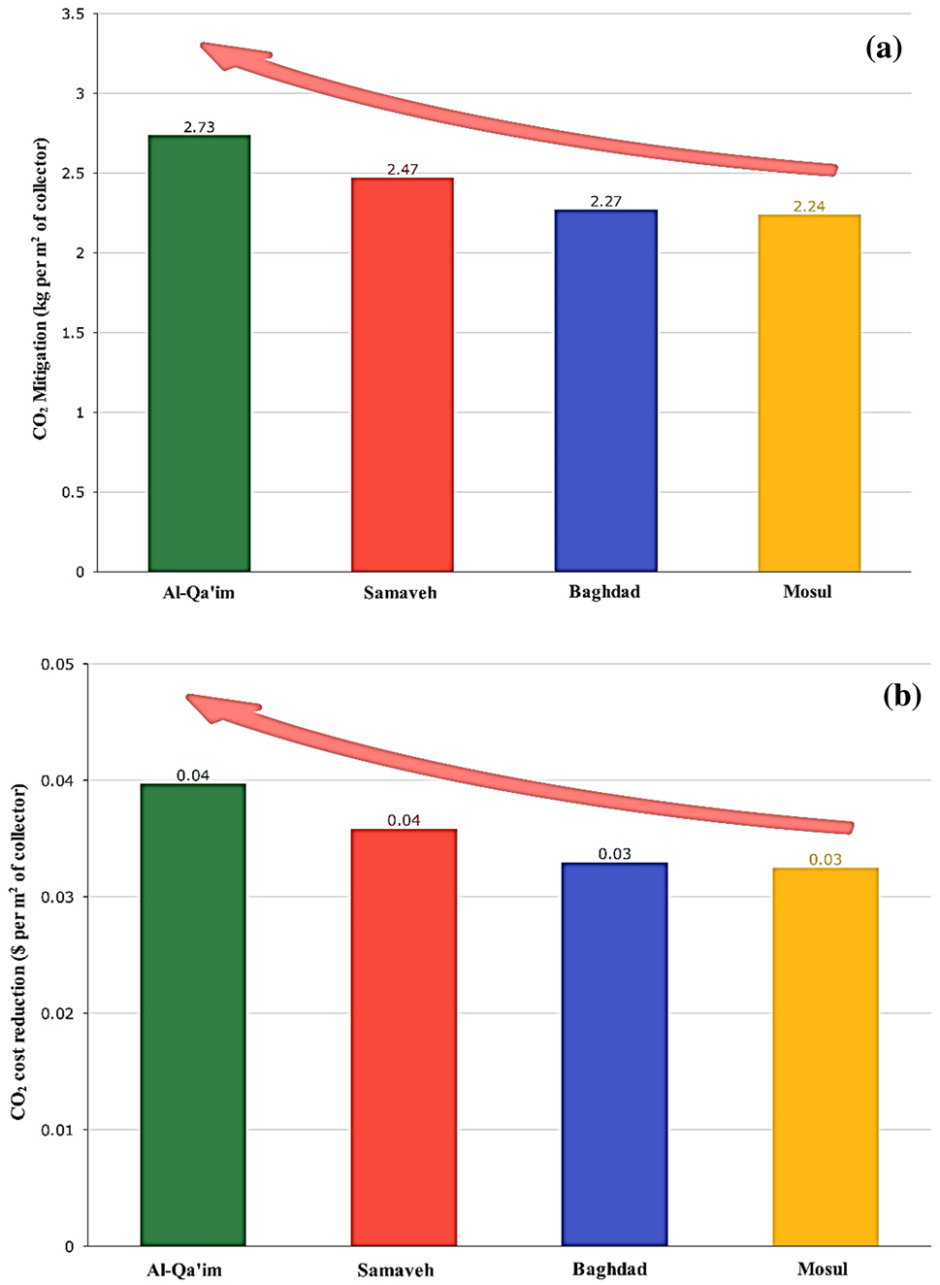
(**a**) The amount and (**b**) Cost of CO_2_ mitigation for different cites.

It should be noted that since the system operates at its peak efficiency in July and during 11–12 h, the results will be determined based on that time for each city. Solar collectors can be analyzed in terms of environmental aspects by calculating CO_2_ mitigation. Figure 16 shows the CO_2_ mitigation and reduction of CO_2_ pollution cost for Baghdad, Samawah, Mosul, and Al-Qa'im cities. As shown in Fig. 16, it is believed that Al-Qa'im has the greatest potential for CO_2_ mitigation (2.73 kg per m^2^ of collector) every year due to the fact that it produces the most useful energy. The ENENEC coefficient defines the estimated cost. There is a maximum reduction of CO_2_ pollution cost for Al-Qa'im every year (0.04 $ per m^2^ of collector).

### Energy and water-saving

Energy and water are the main concerns of today’s life especially in countries experiencing energy and water deficiencies. In this regard, the embodied energy and water in domestic and industrial structures must be investigated. Steel and glass are common substances used in direct absorption by solar collectors at the ratio of 3:1. Steel and glass material contains 32 and 15.9 MJ/kg of embodied energy, as well as 98.64m^3^/kg and 8.24m^3^/kg of embodied water, respectively^43^. Hence, the embodied energy and water can be estimated by calculating the solar collector size:

24 $${\mathrm{A}}_{\mathrm{c}}=\frac{\dot{\mathrm{m}} {\mathrm{C}}_{\mathrm{p}} \left({\mathrm{T}}_{\mathrm{out}}-{\mathrm{T}}_{\mathrm{in}}\right)}{\mathrm{I }{\upeta }_{\mathrm{th}}}$$

**Figure 17:**
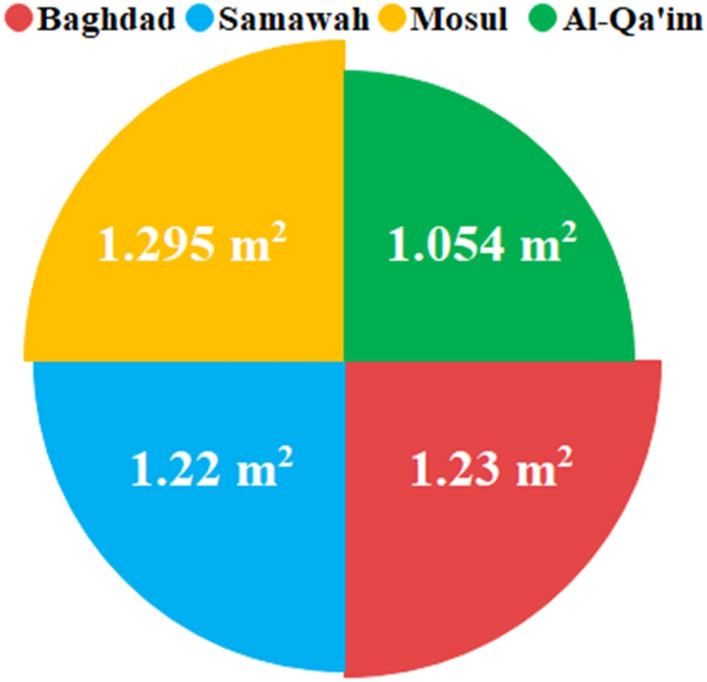
Solar collector size in the different climatic zones.

According to Eq. (24), solar radiation intensity and thermal efficiency of four cities are inversely related to the size of the collector for the same energy received. The average thermal efficiencies of Baghdad, Samawah, Mosul, and Al-Qa'im were 62.96, 63.69, 62.07, and 62.97%, respectively. Figure 17 shows the solar collector size for the same amount of energy received. As expected, Al-Qa'im needs a smaller collector size of 1.054m^2^ due to its higher solar irradiation and thermal efficiency.

**Figure 18:**
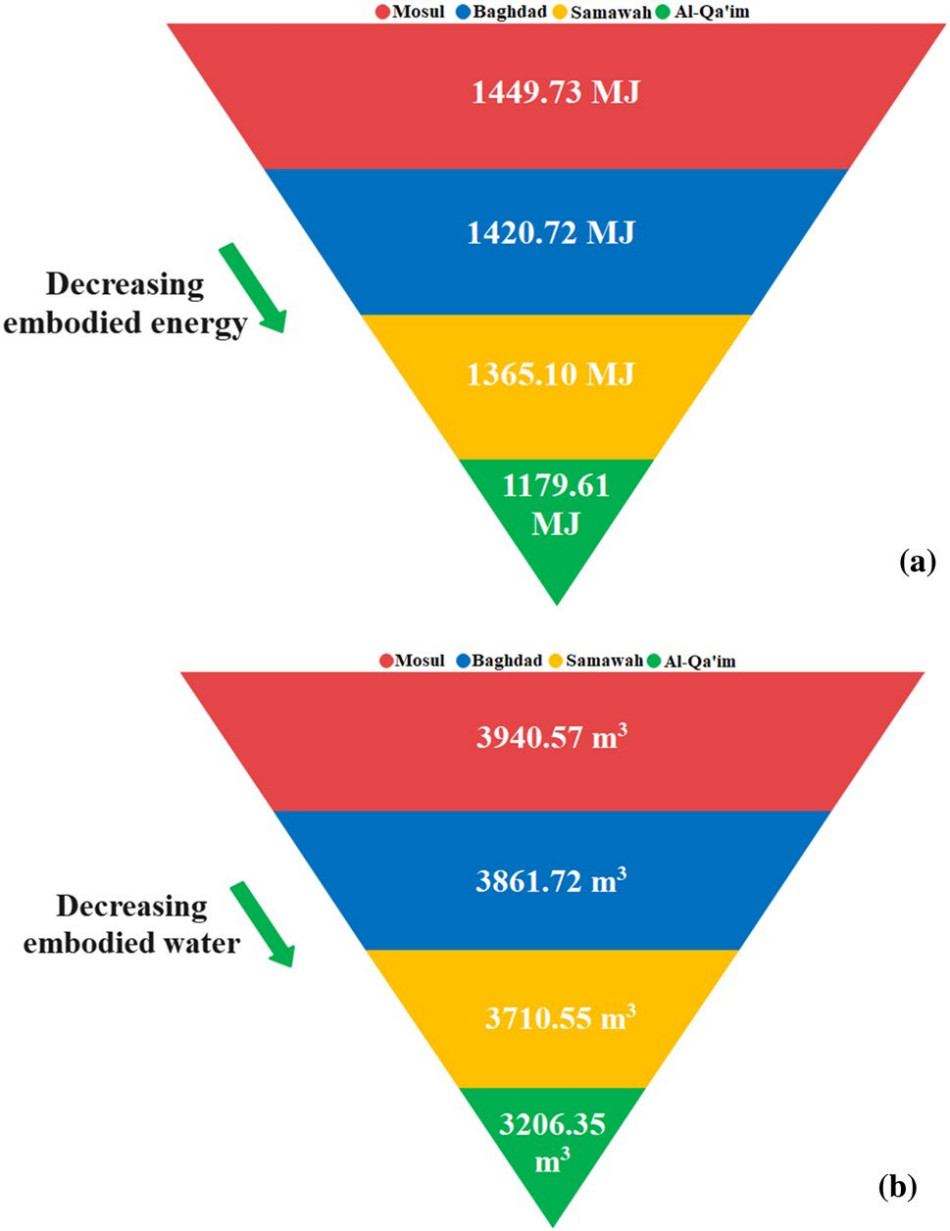
The solar collector's (**a**) embodied energy (**b**) embodied water.

Using the collector size, the embodied energy and water of the solar collector can be determined (see Fig. 18). The solar collectors at Mosul, Baghdad, Samawah, and Al-Qa'im contained embodied energies of 1449.73, 1420.72, 1365.10, and 1179.61 MJ, respectively, as well as embodied waters of 3940.57, 3861.72, 3710.55, and 3206.35 m^3^. Regarding the smaller collector sizes in Al-Qa'im and Samawah, they can save considerably more water and energy.

## Conclusions

**Figure 19:**
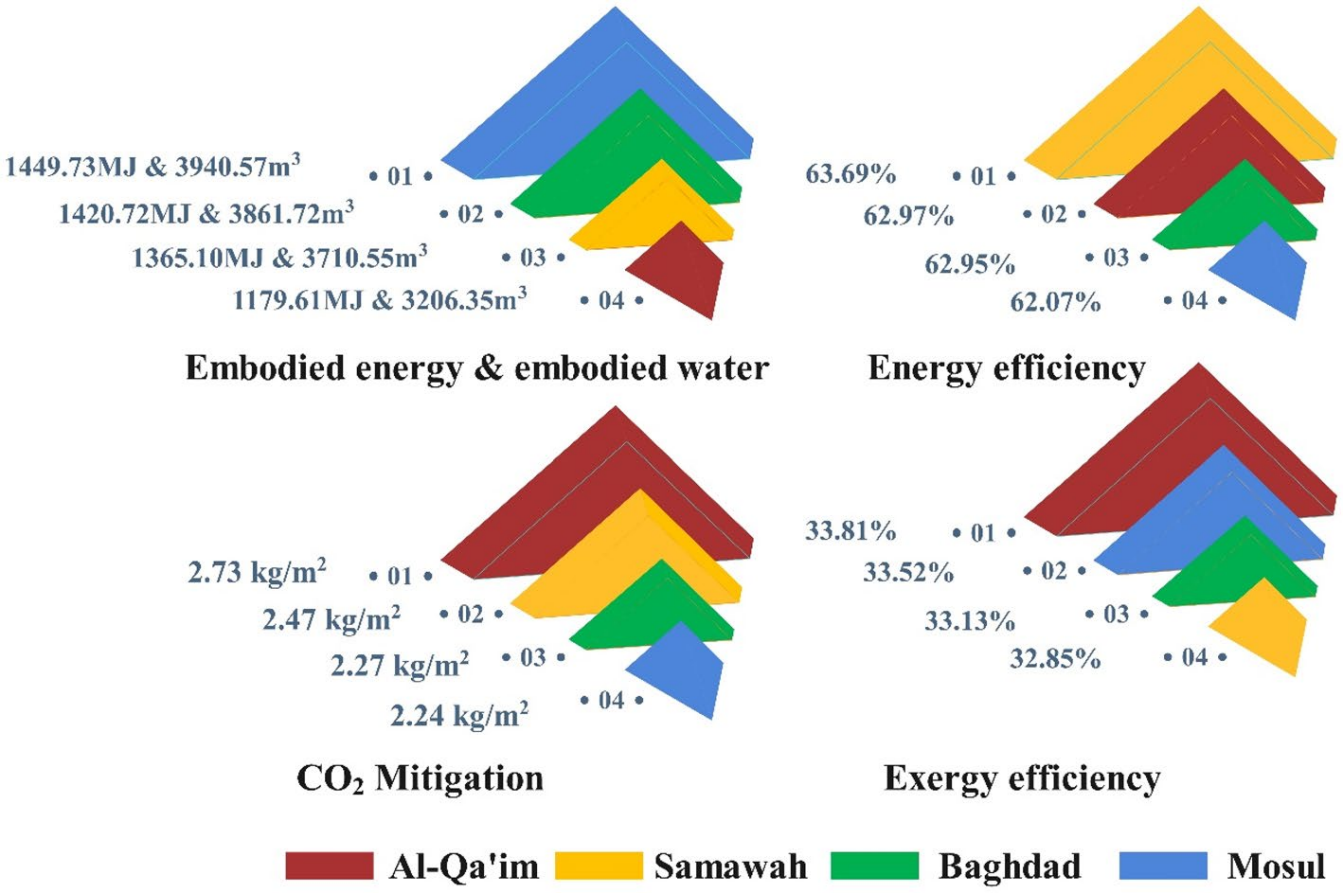
Summary of comparison of the cities.

Energy supply and environmental protection by reducing pollutants are among the main challenges these days. As a clean and sustainable source, solar energy is capable of generating thermal and electrical power. The current study explores solar collector performance considering climate change in terms of energy, exergy, and environmental impacts. To this end, a numerical model was developed and confirmed by experimental findings. Modeling results, along with geometric and optical characteristics of the collector, were derived from information about four cities of Baghdad, Samawah, Mosul, and Al-Qa'im as representative of Iraqi climates. A number of factors, including energy, efficiency, environmental impact, and water-energy nexus, will be taken into consideration when evaluating the collector's performance. These results can be used to determine the most suitable climate for solar energy systems. Figure 19 summarizes these discussions. Energy analysis introduces Samawah as the most suitable climate for a solar collector system with 66.5% energy efficiency, but Al-Qa’im is appropriate in terms of energy received and outlet temperature (599.75 Wh/m^2^ and, 326.84 K, respectively). Exergy analysis introduces Al-Qa’im as the most suitable climate for a solar collector system with an exergy efficiency of 36.21%. Environmental-economic analysis introduces Al-Qa'im as the most suitable climate for a solar collector with CO_2_ mitigation of 2.73 kg per m^2^ of collector. Regarding the high thermal efficiency and solar radiation intensity of Al-Qa’im and Samawah, they can save significantly higher amounts of water and energy.

In general, the efficiency of collectors is affected by the climate. The findings of this research can contribute to the design and exploitation of solar energy systems to determine the appropriate climate for the construction of the system and establish effective environmental policies.

## Data Availability

The datasets used and/or analysed during the current study available from the corresponding author on reasonable request.

## List of symbols

A: Area of the collector (m^2^)
${\mathrm{C}}_{\mathrm{p}}$: Specific heat capacity (J/kg K)
D: Diameter (m)
$$\dot{\mathrm{Ex}}$$: Exergy rate (W)
F: Focal length (m)
$$\mathrm{G}$$: Collector width (m)
h: Heat transfer coefficient $(\frac{\mathrm{W}}{{\mathrm{m}}^{2}\mathrm{ K}})$
$$\mathrm{I}$$: Sun irradiance $$\left(\mathrm{W}/{\mathrm{m}}^{2}\right)$$
$\mathrm{K}$: Incident angle modifier
L: Collector length (m)
Pr: Prandtl number
Re: Reynolds number
r_m_: Mirror reflectance
T: Temperature ($$^\circ{\rm C}$$)
t: Time (s)
u: Velocity (m/s)
$\dot{\mathrm{V}}$: Nanofluid flow rate (LPH)
$$\dot{\mathrm{W}}$$: Power (W)
X: End loss factor
$$\upeta$$: Efficiency
$$\uptheta$$: Incident angle
$\sigma$: Stefan-Boltzmann constant (W/m^2^ K^4^)
λ: Thermal conductivity $$(\frac{\mathrm{W}}{\mathrm{m K}})$$
$$\mathrm{\alpha }$$: Absorption coefficient
$\mu$: Dynamic viscosity (Kg/m s)
$$\upvarepsilon$$: Emittance
$$\uprho$$: Density (Kg/m^3^)
$$\uptau$$: Transmittance
$$\gamma$$: Tracking error

## Subscripts

abs: Absorbed
air: Ambient air
bf: Base fluid
c: Cover
f: Heat transfer fluid
in: Inlet
nf: Nanofluid
opt: Optical
out: Outlet
sa: Surrounding air
s: Sun
t: Tube

## Guidance

${\dot{\mathrm{Q}}}_{\mathrm{t}-\mathrm{c}}^{\mathrm{conv}}$: Convection heat transfer between glass tube & cover
${\dot{\mathrm{Q}}}_{\mathrm{f}-\mathrm{abs}}$: Rate of radiation absorbed by the fluid
${\mathrm{h}}_{\mathrm{t}-\mathrm{c}}$: Heat transfer coefficient between two cylinders
DAPTC: Direct absorption parabolic trough collector
